# Genome-wide DNA methylation pattern in whole blood of patients with Hashimoto thyroiditis

**DOI:** 10.3389/fendo.2023.1259903

**Published:** 2023-11-24

**Authors:** Zheng Zhou, Jinjin Liu, Yun Chen, Bingxuan Ren, Siyuan Wan, Yao Chen, Yanhong He, Qiuyang Wei, Haiyan Gao, Lixiang Liu, Hongmei Shen

**Affiliations:** ^1^ Disorders Control, Centre for Endemic Disease Control, Chinese Centre for Disease Control and Prevention, Harbin Medical University, Harbin, Heilongjiang, China; ^2^ Key Laboratory of Etiology and Epidemiology, National Health Commission & Education Bureau of Heilongjiang Province, Harbin Medical University, Harbin, Heilongjiang, China; ^3^ Heilongjiang Provincial Key Laboratory of Trace Elements and Human Health, Harbin Medical University, Harbin, Heilongjiang, China; ^4^ Department of Preventive Medicine, Qiqihar Medical University, Qiqihar, Heilongjiang, China; ^5^ First Clinical Medical Department, Heilongjiang University of Chinese Medicine, Harbin, Heilongjiang, China; ^6^ Second Department of Endocrinology, The First Affiliated Hospital of Heilongjiang University of Chinese Medicine, Harbin, China; ^7^ Department of Clinical Laboratory, The Sixth Affiliated Hospital of Harbin Medical University, Harbin, China

**Keywords:** Hashimoto thyroiditis, DNA methylation, epigenetics, autoimmune endocrinopathies, SLFN12

## Abstract

**Background:**

Hashimoto thyroiditis (HT), a prevalent autoimmune disorder, is not yet thoroughly understood, especially when it comes to the influence of epigenetics in its pathogenesis. The primary goal of this research was to probe the DNAm profile across the genome in the whole blood derived from patients suffering from HT.

**Method:**

Using the Illumina 850K BeadChip, we conducted a genome-wide DNAm assessment on 10 matched pairs of HT sufferers and healthy individuals. Genes with differential methylation (DMGs) were identified and underwent functional annotation via the databases of Gene Ontology and Kyoto Encyclopedia of Genes and Genomes. The transcriptional significance of potential epigenetic biomarker genes was corroborated through qRT-PCR.

**Results:**

The DNAm profiling across the genome indicated an overall reduction in methylation in HT subjects in comparison with their healthy counterparts. We detected 283 DMPs (adjusted P < 0.05 and |Δβ| > 0.1), among which 152 exhibited hypomethylation and 131 demonstrated hypermethylation. Further analysis exposed a noteworthy concentration of hypermethylated DMPs in the 3´UTR, North Shore, and CpG islands, while there was a significant decrease in the Open Sea (all P < 0.001). The 283 DMPs were broadly distributed from chromosome 1 to 22, with chromosome 6 harboring the most DMPs (n = 51) and chromosome 12 carrying the most DMGs (n = 15). The *SLFN12* gene, which presented with extreme hypomethylation in its promoter DMPs among HT patients, was identified as the epigenetic marker gene. Consequently, the *SLFN12* mRNA expression was markedly upregulated in HT, displaying a negative relationship with its methylation levels. The area under curve (AUC) value for the *SLFN12* gene among HT patients was 0.85 (sensitivity: 0.7, specificity: 0.7), a significant difference compared with healthy controls. The methylation levels of all DMPs in *SLFN12* gene were negatively correlated with TSH and one CpG site (cg24470734) was positively assocciated with FT_4_.

**Conclusion:**

This investigation presents an initial comprehensive DNAm blueprint for individuals with HT, which permits clear differentiation between HT subjects and normal controls through an epigenetic lens. The *SLFN12* gene plays a pivotal role in the onset of HT, suggesting that the methylation status of this gene could serve as a potential epigenetic indicator for HT.

## 1 Introduction

Currently, Hashimoto thyroiditis (HT) is recognized as the predominant autoimmune disorder of the endocrine system, standing as the leading cause of hypothyroidism. It’s distinguished by the presence of antibodies that target thyroid antigens and the lymphocytic penetration of the thyroid gland (1). The onset of HT can be instigated by both genetic predisposing factors and influences from the environment. Twin studies have allowed us to estimate that genetic elements account for 70%-80% of the susceptibility to HT (2, 3). As a result, it’s of utmost importance to pinpoint the genes that possess a genetic susceptibility impacting the initiation and progression of HT through whole-genome sequencing.

In recent times, the potential implications of epigenetics in thyroid-related disorders have begun to gain significant interest. Epigenetics, defined as inheritable modifications that impact gene expression without inducing alterations to the DNA sequence, serves to elucidate the possible correlations among genetic elements, environmental influencers, and the development of HT (4). Of the various epigenetic mechanisms, DNA methylation (DNAm) has been the subject of extensive investigation. DNAm entails the integration of a methyl group into a cytosine unit in a cytosine-phosphate-guanine (CpG) dinucleotide sequence, enabling further regulation of gene expression by impeding the attachment of transcription factors to DNA (5). Multiple research efforts have delved into DNAm segments of HT susceptibility genes. For instance, it was discovered that DNAm in the *ICAM-1* promoter area was diminished in HT patients and exhibited a negative correlation with thyrotropic immunoglobulin concentration, which ultimately influenced the adhesion of cytotoxic T cells to their targets (6). In a separate study, significantly elevated levels of DNAm were identified in the promoter region of *PTPN22* in young patients with HT, relative to controls; this verified the role of *PTPN22* as an epigenetically determined susceptibility gene for thyroid autoimmunity (7).

To conclude, it’s evident that alterations in DNAm hold a significant correlation with HT. While many studies have explored the potential changes in methylation levels of susceptibility genes amongst HT patients, to our understanding, no prior research has published a genome-wide DNAm study specifically involving HT patients sourced from the general population. Hence, in the present research, we conducted a comprehensive DNAm investigation using whole blood specimens from both HT patients and healthy individuals. Our study’s objective was to identify the crucial differentially methylated genes (DMGs) through the means of bioinformatics analysis and subsequently pinpoint the epigenetic biomarker gene linked with HT, thereby furnishing pivotal molecular insights into HT’s pathogenesis.

## 2 Materials and methods

### 2.1 Study participants

**Figure 1:**
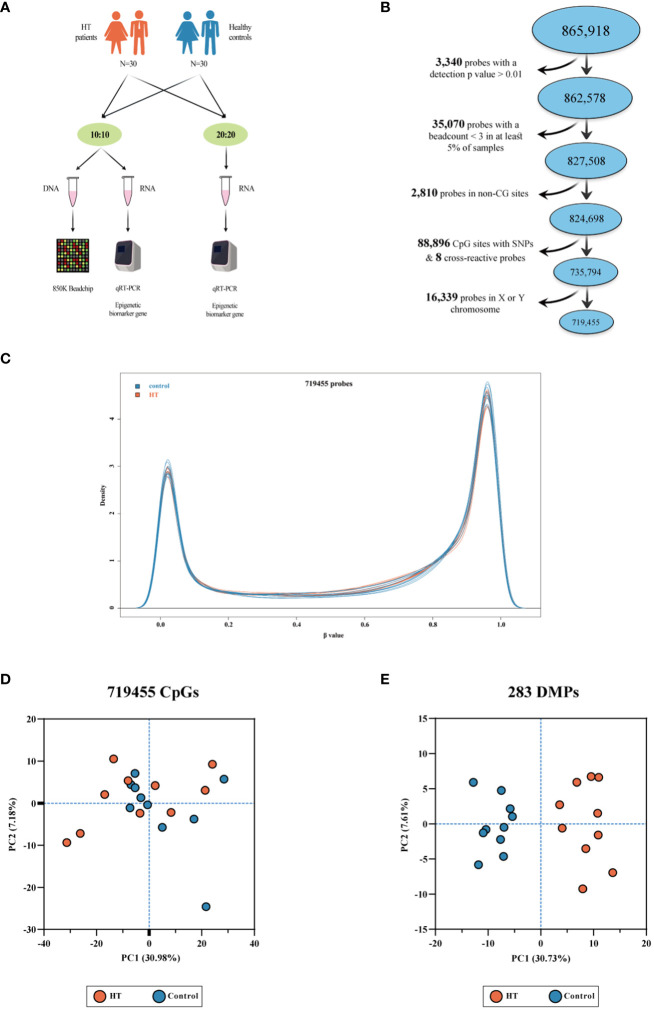
A series of depictions pertaining to the 850K BeadChip, the density plot, and the PCA plot. **(A)** Presents the experimental procedure. **(B)** Illustrates the quality assurance and data preprocessing measures adopted for the 850K BeadChip. **(C)** Depicts a density plot. Beta values express the levels of methylation in both HT patients and healthy controls. **(D)** Showcases a PCA plot grounded on the 719,455 CpG sites present in the 850K BeadChip, with no clear demarcation discernible between HT patients and the controls. **(E)** Reveals a PCA plot grounded on 283 differentially methylated positions (DMPs). The plot shows clear clustering differences between HT patients and healthy controls.

In the Shandong Province of China, June 2019 saw the involvement of a collective group of 30 paired matches, comprising of HT-affected subjects and healthy control individuals. The diagnosis of HT was effectively established by an experienced endocrinologist, using two key indicators: 1. The existence of serum thyroid peroxidase antibodies (TPOAb), thyroglobulin antibodies (TgAb), or a combination of both; 2. Apparent signs of goiter, changes in echogenicity, or multiple hypoechoic regions as determined by thyroid ultrasound tests. On the other hand, the criteria for selection of control participants included: 1. Healthy individuals of similar sex, age, locale, and BMI to the HT-affected subjects; 2. Absence of personal or familial histories of autoimmune disorders or other thyroid complications, no chronic or acute illnesses, no record of long-term thyroid-related medication or hormone use, and non-pregnant status; 3. A clean bill of health in terms of goiter; negative for both TgAb and TPOAb; normal thyroid function indicators, and no irregularities spotted in thyroid ultrasound examinations. Blood samples were drawn from all participants and kept in a -80°C storage for later examinations. As shown in **Figure 1A**, out of the 30 pairs, 10 were selected for the genome-wide DNAm analysis while the entire group was used for mRNA expression examination of the epigenetic biomarker gene. The research upheld the standards set in the Declaration of Helsinki and received approval from the Ethics Review Committee of Harbin Medical University (No. hrbmuecdc20200320). The participants provided their consent by signing the necessary forms.

### 2.2 Assessment of thyroid function and detection of thyroid antibody

Measurements of free triiodothyronine (FT_3_), free thyroxine (FT_4_), thyroid stimulating hormone (TSH), TgAb, and TPOAb were obtained through the application of chemiluminescence immunoassay techniques, a service facilitated by Siemens Inc., Tarrytown, NY, USA. The defined standard values for these measures were set as follows: for FT_3_, an acceptable range was considered to be 3.1 pmol/L to 6.8 pmol/L; FT_4_ standard value resided between 11.5 pmol/L and 22.7 pmol/L; TSH was designated as normal if it fell between 0.27 mIU/L and 4.20 mIU/L. To detect thyroid antibodies, benchmarks were set with the normal threshold for TgAb and TPOAb each maintained within the bounds of 0–60 U/mL.

### 2.3 Genome-wide DNAm measurement

The TIANGEN Extraction Kit (TIANGEN, Beijing, China) was employed for the procedure of genome-wide DNA extraction. The purity and concentration of the harvested DNA were subsequently confirmed with the assistance of the Nanodrop 2000 instrument (Thermo Fisher, Waltham, USA). Following this, we processed 500 ng of DNA from each specimen for bisulfite conversion, a procedure facilitated by the EZ DNA Methylation Kits (Zymo Research, California, USA). Post conversion, the resulting products were processed further using the Illumina Infinium Human Methylation 850K BeadChip (Illumina Inc, California, USA), in strict accordance with the manufacturer’s directives.

### 2.4 Genome-wide DNAm BeadChip analysis

The BeadChip was scanned using an Illumina iScan instrument. The raw intensity data, also known as IDAT, was loaded into Rstudio for further analysis. Using the Chip Analysis Methylation Pipeline (ChAMP) package (2.14.0), the data underwent preprocessing, normalization, and group comparison processes (8). To identify confounding factors, singular value decomposition analysis was executed. To elaborate, the minfi package (1.30.0) was used for preprocessing the raw data, and technical variations were normalized with SWAN (9). Certain probes were excluded from the analysis based on the following specific criteria: 1. Any CpG probes that had a detection P ≥ 0.01 in one or more samples; 2. probes with fewer than three beads in 5% of the samples; 3. non-CpG probes; 4. cross-reactive probes and probes associated with single nucleotide polymorphisms (SNPs); 5. probes located on the X or Y chromosomes.

### 2.5 Differential methylation position analysis and the distribution analysis of DMPs

A β value was utilized to signify the methylation extent for each CpG site, with the scale ranging from 0 (0% methylation) to 1 (100% methylation of a specific CpG dinucleotide). The Δβ was computed by deducting the β value of the control group from that of the HT cases. A CpG site with Δβ > 0 was deemed as hypermethylated, whereas hypomethylation was assigned when Δβ < 0. Differential methylated positions (DMPs) were discerned by contrasting mean β values in the HT group with those in the normal group for a given CpG site through ChAMP analysis (10). We established DMP criteria by setting a P value of less than 0.05 and a β value discrepancy between groups greater than 0.1 (|Δβ| > 0.1). The DMPs were then segregated into distinct groups based on various gene regions: TSS1500, TSS200, 5’UTR, 1stExon, gene body, 3’UTR, and intergenic region (IGR). In accordance with different CpG island regions, the DMPs were also divided into: North Shelf, North Shore, CpG Island, South Shore, South Shelf, and Open Sea, wherein shores were 0–2 kb away from a CpG island and shelves were 2–4 kb distant. The entire array data processing and analysis were executed in Rstudio.

### 2.6 Gene ontology and Kyoto encyclopedia of genes and genomes enrichment

Every identified DMP was matched to its respective DMGs. To delve into the prospective biological roles of these DMGs, we employed the R package clusterProfiler for conducting GO analysis and KEGG pathway examination. To identify the significantly enriched GO terms and pathways, we set a P value of less than 0.05 as the cut-off criterion.

### 2.7 Selection of epigenetic biomarker gene

Subsequently, we embarked on a mission to further identify potential epigenetic biomarker genes linked to HT. We adopted a strategic set of screening criteria for DMGs previously screened through Illumina 850K BeadChip. The rules for potential epigenetic biomarker genes included: 1. DMGs situated within promoter regions (namely, TSS1500, TSS200, 5′UTR, and 1^st^Exon); 2. DMGs with strikingly high or low methylation levels (with a β value exceeding 0.7 or under 0.3); 3. DMGs possessing at least three corresponding DMPs.

### 2.8 Quantitative real-time PCR

RNA extraction was performed from total blood samples utilizing RNAiso Plus (Takara, Dalian, China). The concentration of RNA was ascertained using the NanoDrop 2000 (Thermo Fisher, Waltham, USA). RNA quality was deemed satisfactory with an OD _260/280_ ratio ranging between 1.8 and 2.0. Each specimen was further processed using a QuantStudio™5 Real-Time PCR system (Thermo Fisher, Waltham, USA). The amplification reaction was set up with the following constituents: 5.0 μL SYBR Green (Roche, Basel, Switzerland), 1.0 μL cDNA, 0.5 μL of each primer (upstream and downstream), and 3.0 μL ddH_2_O. The PCR protocol was as follows: 1. initial denaturation at 95°C for 10 min; 2. forty cycles of 95°C for 15s and 60°C for 1 min; 3. melt curve stage at 95°C for 15s, 60°C for 1 min, and 95°C for 15s. The primers were designed as such: *SLFN12-F: 5′-TGG CCT CTT TTG GAA TGG CA-3′; SLFN12-R: 5′-CAA GCA GCC CAG ATC CAC AGA CC-3′; β-actin-F: 5′-CCT TCC TGG GCA TGG AGT CCT G-3′; β-actin-R: 5′-GGA GCA ATG ATC TTG ATC TTC-3′*. The 2^-ΔΔCt^ method facilitated the analysis of mRNA levels of the epigenetic biomarker gene.

### 2.9 Statistical analysis

Data from the 850K BeadChip, as well as GO and KEGG, underwent statistical evaluation in the RStudio software (Version 1.2.1335) (http://www.rstudio.com/) within an R coding environment (version 3.6.0) (https://www.R-project.org). Participant demographic details and thyroid functionality data were inputted into Microsoft Office Excel 2016 and analyzed statistically using the SPSS 23.0 software. The Kolmogorov–Smirnov approach was used to test for normal data distribution. Data adhering to normal distribution are displayed as mean ± standard deviation (mean ± SD), and mean discrepancies were examined using student’s t-test. Non-normally distributed data are expressed through the median along with the 25^th^ and 75^th^ percentiles. Comparisons among various groups were executed using the Mann–Whitney U test. The chi-square (χ^2^) test was applied for comparing rates among groups with categorical results. The associations between relative mRNA expression and the methylation levels of *SLFN12* were scrutinized via Pearson correlation analysis. The diagnostic value and the cutoff point were ascertained through receiver operating characteristic (ROC) curve analysis. Correlation between the DNAm levels of 5 DMPs in *SLFN12* and variables (age, FT_3_, FT_4_, and TSH) was assessed through Spearman’s rank or Pearson correlation analyses. A p-value of less than 0.05 was taken to be statistically significant.

## 3 Results

### 3.1 Global DNAm profile of HT patients

**Table 1** delineates the demographic attributes and thyroid functions of 10 pairs consisting of Hashimoto’s thyroiditis (HT) patients and healthy control subjects analyzed using the 850K BeadChip. All the subjects classified under the HT group were in compliance with the HT diagnostic criteria. **Supplementary Table 1** provides the demographic details for 30 pairs of HT patients and control subjects and their thyroid functions for validation. The BeadChip detected a total of 865,918 CpG sites for future manipulation. Applying the selection process as described in our materials and methods section, a total of 719,455 CpG sites were retained for subsequent scrutiny (**Figure 1B**). Following this, normalization of data and quality control was performed on these 719,455 CpG sites. As observable from the density plot (**Figure 1C**), the methylation level distribution of HT patients and their healthy counterparts showed considerable similarity after the aforementioned procedure. We then executed principal component analysis (PCA) on the 719,455 CpG sites (**Figure 1D**). The outcome demonstrated no clear demarcation between the HT patients and healthy control subjects.

**Table 1:** The basic information of HT patients and healthy controls in 850K.

Group	Sample	Sex	Age (years)	BMI (kg/m2)	TSH (mIU/L)	FT_3_ (pmol/L)	FT_4_ (pmol/L)	TPOAb(IU/ml)	TgAb(IU/ml)	Thyroid ultrasound
Control	C1	Female	30	25.71	3.59	5.95	17.18	11.74	8	Normal
	C2	Female	32	23.92	0.51	4.71	19.73	9.21	7	Normal
	C3	Female	34	25.39	1.58	4.46	12.97	9.73	7	Normal
	C4	Female	39	21.51	2.51	4.87	16.53	7.48	6	Normal
	C5	Female	42	25.39	1.75	3.97	20.54	<5.00	6	Normal
	C6	Female	46	22.83	3.78	4.72	17.57	11.20	6	Normal
	C7	Female	46	19.81	2.90	4.98	18.75	20.19	5	Normal
	C8	Female	46	24.09	2.32	4.74	16.09	7.50	6	Normal
	C9	Female	48	22.03	0.76	4.10	16.61	11.28	8	Normal
	C10	Female	54	19.83	3.03	3.87	15.16	8.55	4	Normal
HT	HT1	Female	30	20.70	100.00 ↑	2.55 ↓	3.54 ↓	>1300.00 ↑	41	Bilateral diffuse thyroid lesions
	HT2	Female	31	25.71	101.45 ↑	5.04	7.60 ↓	>1300.00 ↑	36	Bilateral diffuse thyroid lesions, abnormal hypoechoic area of left thyroid, cystic nodule of the right thyroid
	HT3	Female	34	23.15	5.84 ↑	4.67	15.11	>1300.00 ↑	39	Bilateral diffuse thyroid lesions and left thyroid nodule
	HT4	Female	40	23.14	6.57 ↑	4.75	17.85	>1300.00 ↑	31	Bilateral goiter with diffuse lesions, cystic and solid nodules of the right thyroid
	HT5	Female	40	20.03	93.49 ↑	2.95 ↓	3.85 ↓	215.10 ↑	18	Bilateral goiter with diffuse lesions
	HT6	Female	42	23.88	14.98 ↑	5.79	10.30 ↓	>1300.00 ↑	32	Goiter with diffuse lesions, calcification in the right thyroid parenchyma, bilateral thyroid nodules, and partial nodules with calcification
	HT7	Female	44	20.96	4.86 ↑	4.64	17.62	>1300.00 ↑	34	Bilateral diffuse thyroid lesions
	HT8	Female	48	24.89	>105.45 ↑	0.40 ↓	0.72 ↓	>1300.00 ↑	29	Bilateral diffuse thyroid lesions
	HT9	Female	51	21.26	4.66 ↑	3.95	13.84	>600.00 ↑	36	Goiter with diffuse lesions
	HT10	Female	54	19.81	8.27 ↑	4.37	17.73	459.10 ↑	53	Bilateral diffuse thyroid lesions

HT, Hashimoto thyroiditis; BMI, body mass index; TSH, thyroid stimulating hormone; FT_3_, free triiodothyronine; FT_4_, free thyroxine; TPOAb, thyroid peroxidase antibodies; TgAb, thyroglobulin antibodies; ↓, indicates lower than the reference ranges; ↑, indicates higher than the reference ranges.

**Figure 2:**
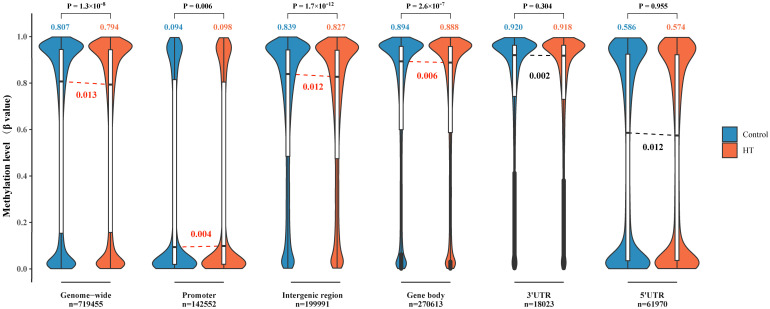
Comparison of DNA methylation levels across various genomic locations in HT patients versus healthy controls. The violin plots reflect DNA methylation at a genomic scale (n = 719,455), within promoters (n = 142,552), spanning intergenic regions (n = 199,991), across gene body regions (n = 270,613), within 3′UTRs (n = 18,023), and 5′UTRs (n = 61,970). The embedded box plots in each violin plot signify the interquartile range, and the red lines delineate a significant statistical deviation between HT patients and the healthy controls. For all the cases, the y-axis denotes the methylation level (β value) on a scale from 0 to 1.

Next, we scrutinized the DNAm levels in various genomic locations in both HT patients and their healthy counterparts (**Figure 2**). First and foremost, the patterns of genome-wide DNAm spanning 719,455 CpG sites exhibited a bimodal distribution. Most CpG sites, in both HT patients and control groups, showed high (β > 0.8) or low (β < 0.2) levels of DNAm. In a general sense, the methylation levels in HT were considerably lower compared to the healthy controls, and this difference was statistically significant (P < 0.001). In the second place, a substantial number of unmethylated CpG sites (β < 0.1) were found distributed within the promoter and 5’UTR region. The promoter region in HT patients displayed higher methylation levels compared to the healthy control group, and this discrepancy was statistically significant (P = 0.006). Last but not least, both IGR and gene body displayed a downward shift in median methylation in HT patients when juxtaposed with healthy controls (both P < 0.001). These outcomes point to the conclusion that the DNAm levels within different gene regions undergo significant changes in HT patients.

### 3.2 DMPs in HT patients

**Figure 3:**
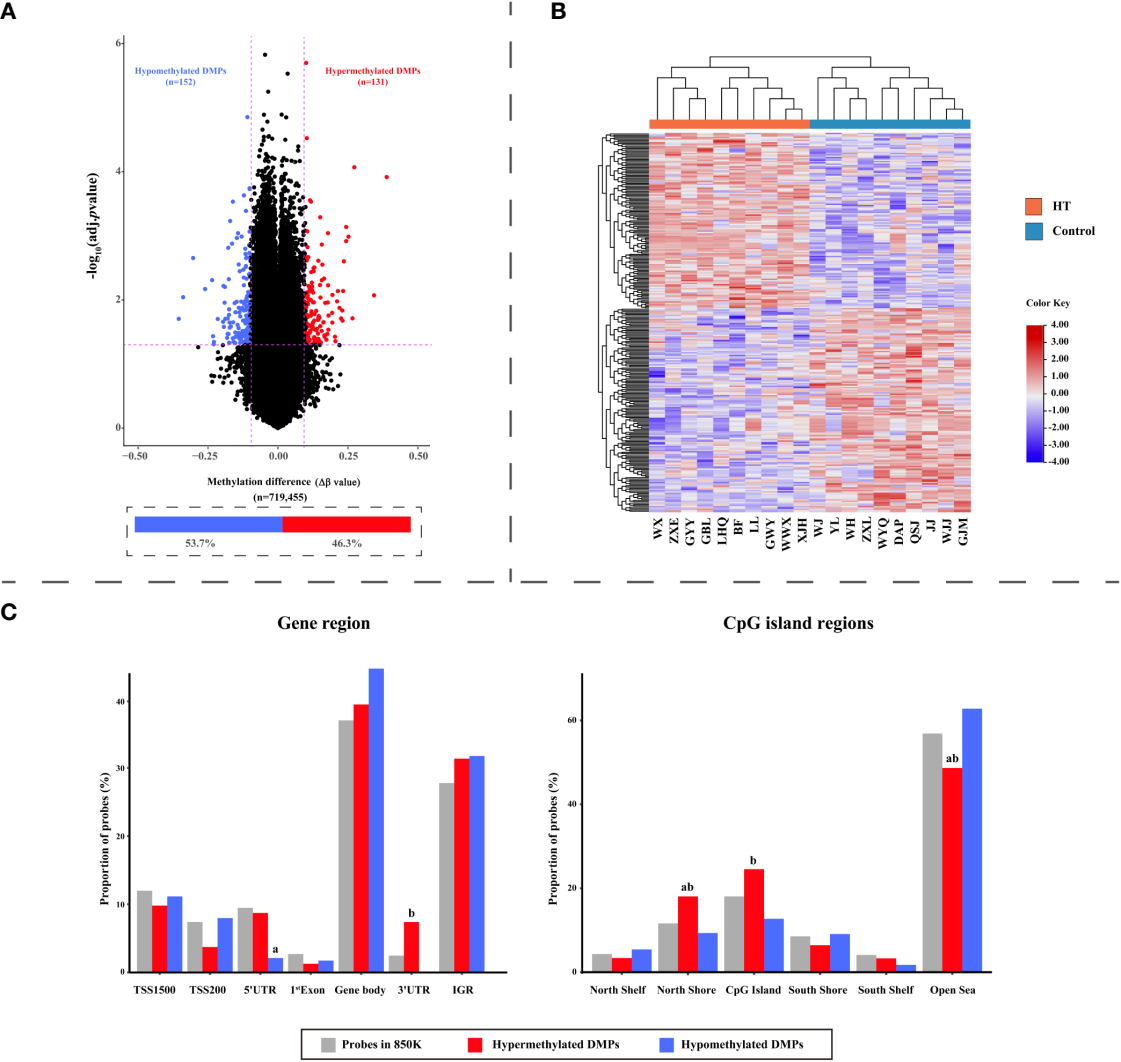
The dispersion of differentially methylated probes (DMPs) in HT patients. **(A)** A Volcano plot visualizing probe-level methylation in HT patients compared to healthy controls. The diagram presents the relationship between Δβ values (along the x-axis) and adjusted P values (-log10 transformed adjusted P values) on the y-axis. Each dot is representative of an individual probe. The demarcations for adjusted P = 0.05 and |Δβ| = 0.1 are indicated by horizontal and vertical dashed lines, respectively. Red and blue dots symbolize hyper- and hypomethylated DMPs, correspondingly. **(B)** Heatmap of the 283 DMPs distinguishing HT patients from healthy controls. Each row stands for a unique DMP and each column symbolizes a participant. The diagram consists of 283 rows (indicative of 283 DMPs) and 20 columns (denoting 10 HT patients versus 10 healthy controls). On the top, red and blue bars represent HT patients and healthy controls, respectively. Color transition from red to blue signifies a shift from hypermethylation to hypomethylation levels. **(C)** Bar charts illustrating the distribution of DMPs with respect to gene region (left panel) and CpG island region (right panel). The total probes in the 850K BeadChip available for analysis (grey; n = 719,455), hypermethylated DMPs (red; n = 131), and hypomethylated DMPs (blue; n = 152) as per gene region and CpG island region. P values were computed by Chi squared tests. ‘a’ suggests the difference is statistically significant compared with probes in the 850K BeadChip; ‘b’ indicates that the difference is statistically significant compared to hypomethylated DMPs.

**Figure 4:**
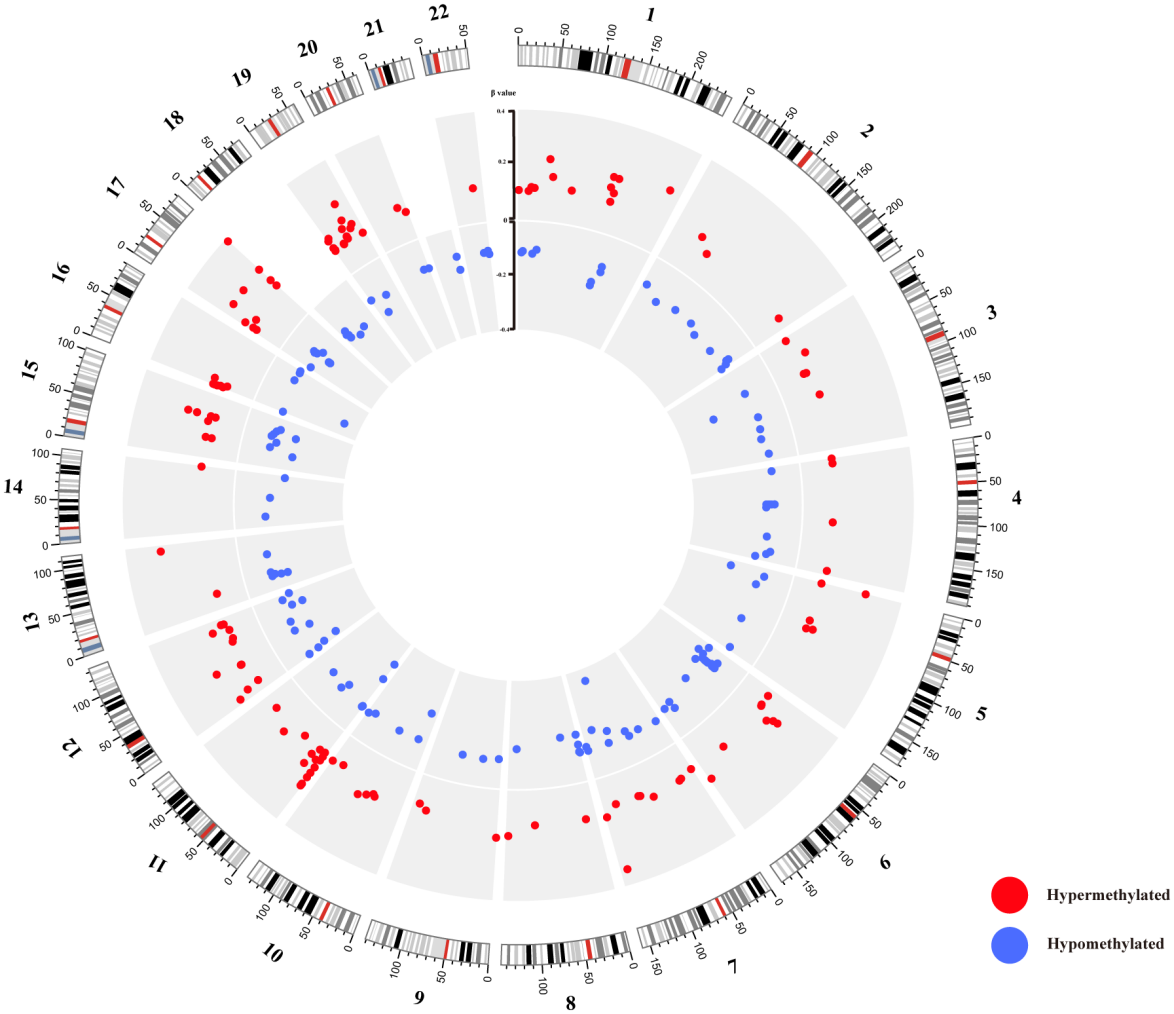
Circos diagram of differentially methylated probes (DMPs). The circos plot delineates the distribution of 283 DMPs across 22 chromosomes; the exterior layer depicts the physical location of each chromosome; the intermediate layer illustrates chromosome segments; the innermost layer signifies the methylation site. Here, red color corresponds to hypermethylation DMPs, while blue represents hypomethylation DMPs.

In contrast to the 10 healthy controls, HT patients revealed a total of 283 differential methylation positions (DMPs) (adjusted P < 0.05, |Δβ| > 0.1). A volcano plot was used to illustrate the DMPs, where it was shown that of the 283 DMPs, 152 (53.7%) demonstrated hypomethylation, and 131 (46.3%) exhibited hypermethylation (**Figure 3A**). The top 10 DMPs showing hypermethylation and hypomethylation were ranked according to |Δβ| and are presented in **Supplementary Table 2**. Utilizing the 283 DMPs, the PCA distinguished two unique clusters separating the HT patients from the healthy controls (**Figure 1E**). The unsupervised hierarchical cluster heatmap of 283 DMPs distinctly segregated HT patients and healthy controls (**Figure 3B**). Each row within the heatmap denotes a CpG probe while each column represents an independent sample. Given the known relationship between the specific genomic location of DMPs and their effect, we then endeavored to determine the genomic distribution of DMPs. The distribution of hypomethylated DMPs in the 5’UTR was significantly lower (2.63%) compared to the probes in 850K BeadChip, whereas the distribution of hypermethylated DMPs in the 3’UTR was significantly increased (6.10%) in comparison to hypomethylated DMPs (both P < 0.001) (**Figure 3C** left panel). As for the CpG island regions, the hypermethylated DMPs were significantly overrepresented in North Shore (16.03%) but notably lower in Open Sea (47.32%) when compared to the probes in 850K BeadChip and hypomethylated DMPs (all P < 0.001). Furthermore, in comparison to hypomethylated DMPs, hypermethylated DMPs were significantly overrepresented in CpG islands (23.7%) (P < 0.001) (**Figure 3C** right panel). The 283 DMPs in HT patients were broadly distributed across chromosomes 1 through 22 (**Figure 4**). Out of the 22 chromosomes, chromosome 6 had the most DMPs (n = 30) and hypomethylated DMPs (n = 22); chromosome 11 had the highest number of hypermethylated DMPs (n = 15). Additionally, chromosome 12 housed the largest number of differentially methylated genes (DMGs) (15 genes). All hypermethylated and hypomethylated DMGs on chromosomes 1 through 22 are listed in **Supplementary Table 3**.

### 3.3 GO functional analysis and KEGG pathway analysis

**Figure 5:**
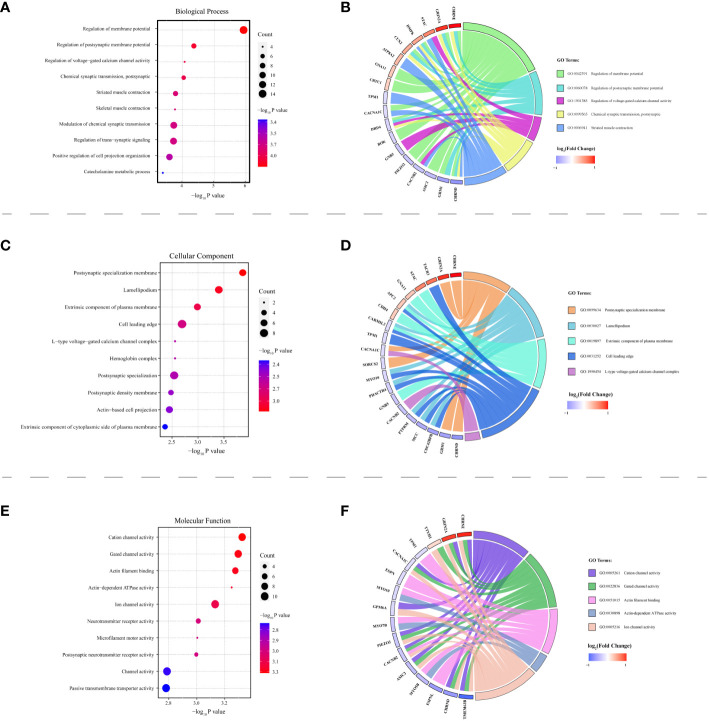
HT-related differentially methylated genes (DMGs) explored via Gene Ontology (GO) analysis. Panels **(A, C, E)** present bubble charts, each showcasing the top 10 enriched terms under biological process (BP), cellular component (CC), and molecular function (MF) respectively. The vertical axis represents the specific GO pathway’s name, while the P-value is plotted on the horizontal axis. The bubble size corresponds to the count of differentially methylated genes participating in that pathway. The color intensity of each bubble relates to the –log10 P-value. Panels **(B, D, F)** feature the GO Chord plots illustrating the association between DMGs and their relevant BP, CC, and MF terms. Various color blocks denote distinct GO terms with the symbols of targeted genes displayed on the chart’s left. The colored lines on the right establish the gene involvement under each GO term. Red blocks denote DMGs with hypermethylation, and blue blocks correspond to hypomethylated DMGs.

**Figure 6:**
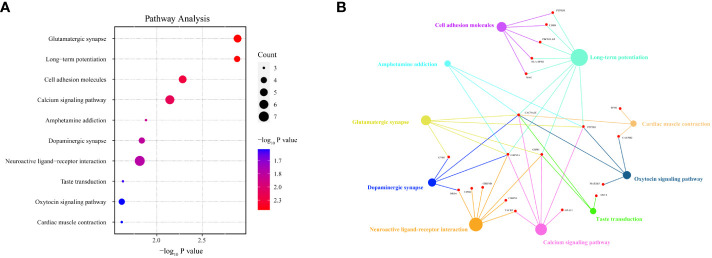
Pathway analysis of HT-related differentially methylated genes (DMGs) based on Kyoto Encyclopedia of Genes and Genomes (KEGG). Panel **(A)** depicts a bubble chart, illuminating the top 10 KEGG-enriched pathways. Panel **(B)** shows that among the top 10 signaling pathways, 20 DMGs are notably enriched. The dot size is indicative of the count of DMGs enriched.

Among the 283 identified DMPs, 147 had biological data available and corresponded to differentially methylated genes (DMGs). A total of 246 biological process (BP) terms were enriched by these DMGs, of which 59 demonstrated significant enrichment (all P < 0.010) (**Supplementary Table 4**). For cellular component (CC) terms, 42 were enriched by all DMGs with 10 being significant (all P < 0.010) (**Supplementary Table 5**). When considering molecular function (MF), 37 terms were enriched by all DMGs and 13 showed significant enrichment (all P < 0.010) (**Supplementary Table 6**). The top 10 terms enriched by BP, CC, and MF are demonstrated in **Figures 5A, C, E** respectively. Concurrently, **Figures 5B, D, F** highlight all the DMGs enriched in the top 5 terms of BP, CC, and MF, respectively. A subset of 18 DMGs emerged in the top 5 BP terms, consisting of 8 hypermethylated and 10 hypomethylated genes. In the top 5 CC terms, a total of 20 DMGs were enriched, composed of 7 hypermethylated and 13 hypomethylated genes. Similarly, in the top 5 MF terms, 16 DMGs were discovered including 3 hypermethylated and 13 hypomethylated genes. A total of 85 pathways were enriched by all genes, with 17 pathways showing significant DMGs enrichment (all P < 0.050) (**Table 2**). Among these pathways, cell adhesion molecules (hsa04514), Th17 cell differentiation (hsa04659), and calcium signaling pathway (hsa04020) were noted for their relevance to autoimmune disease. The top 10 pathways significantly enriched by KEGG of the 147 DMGs are shown in **Figure 6A**, while **Figure 6B** highlights the 20 DMGs enriched in these top 10 signaling pathways.

**Table 2:** 17 significant pathways of KEGG analysis.

Pathway ID	Description	P value	Gene ID
hsa04724	Glutamatergic synapse	0.001	CACNA1C/GNB5/GRIN2A/GRM1/PPP3R1
hsa04720	Long-term potentiation	0.001	CACNA1C/GRIN2A/GRM1/PPP3R1
hsa04514	Cell adhesion molecules	0.005	CDH4/HLA-DPB1/MAG/PDCD1LG2/PTPRM
hsa04020	Calcium signaling pathway	0.007	CACNA1C/GNA11/GRIN2A/GRM1/PPP3R1/TACR3
hsa05031	Amphetamine addiction	0.013	CACNA1C/GRIN2A/PPP3R1
hsa04728	Dopaminergic synapse	0.015	CACNA1C/DRD4/GNB5/GRIN2A
hsa04080	Neuroactive ligand-receptor interaction	0.015	CHRND/CHRNE/DRD4/GRIN2A/GRM1/TACR3/VIPR2
hsa04742	Taste transduction	0.024	ASIC2/CACNA1C/GRM1
hsa04921	Oxytocin signaling pathway	0.024	CACNA1C/CACNB2/MAP2K5/PPP3R1
hsa04260	Cardiac muscle contraction	0.024	CACNA1C/CACNB2/TPM1
hsa04540	Gap junction	0.025	GNA11/GRM1/MAP2K5
hsa05410	Hypertrophic cardiomyopathy	0.027	CACNA1C/CACNB2/TPM1
hsa05150	Staphylococcus aureus infection	0.031	C1R/CFD/HLA-DPB1
hsa05414	Dilated cardiomyopathy	0.031	CACNA1C/CACNB2/TPM1
hsa04713	Circadian entrainment	0.032	CACNA1C/GNB5/GRIN2A
hsa04659	Th17 cell differentiation	0.042	HLA-DPB1/PPP3R1/SMAD3
hsa04725	Cholinergic synapse	0.047	CACNA1C/GNA11/GNB5

### 3.4 Hypomethylation of *SLFN12* gene, an epigenetic biomarker gene for HT

**Figure 7:**
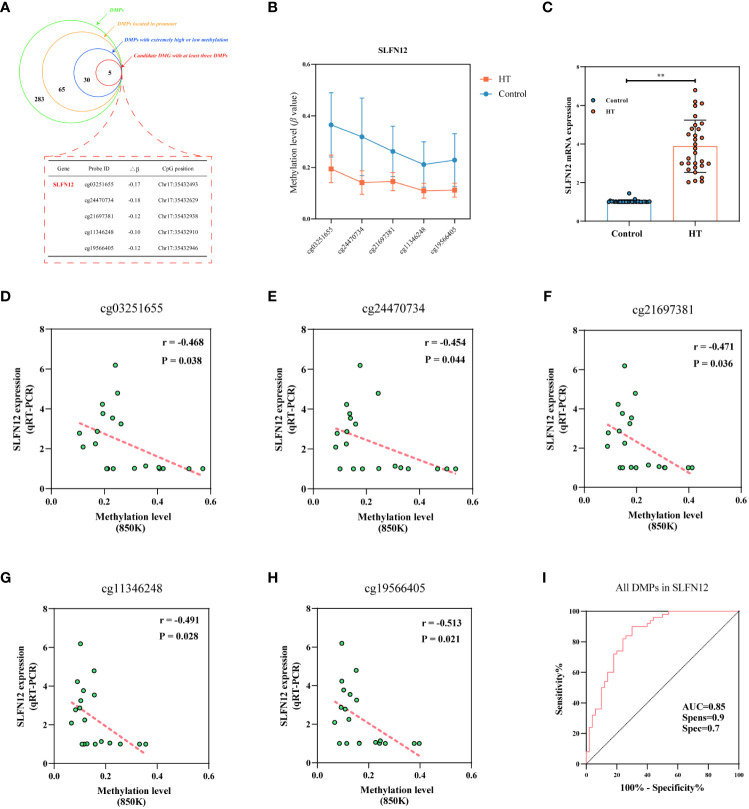
Unveiling the epigenetic marker gene in HT. Panel **(A)** represents a schematic demonstrating the number of DMPs discerned in each stage during the screening progression of the epigenetic marker gene, with SLFN12 singled out for verification, as denoted by the red dotted rectangle. Panel **(B)** illustrates methylation states at five distinct locations of the SLFN12 gene among HT patients versus healthy controls (mean ± SD). Panel **(C)** presents SLFN12 mRNA expression levels, where ** denotes P < 0.001. Panels **(D–H)** exhibit the correlation established between DNA methylation and relative mRNA expression across five specific SLFN12 gene sites. Panel **(I)** presents the ROC curve tailored for SLFN12.

The procedure for screening DMGs potentially linked to the onset of HT, with the aim of identifying epigenetic biomarker genes, is presented in **Figure 7A**. Initially, our focus was confined to the DMGs found on the promoter. Sixty-five DMPs resided in the promoter region, corresponding to a total of 46 DMGs (Refer **Supplementary Table 7**). To further refine the pool of DMGs, we took into account the methylation level of each DMP, selecting only those DMPs with an absolute high methylation level (β > 0.7) or an absolute low methylation level (β < 0.3). This strategy resulted in 30 DMPs that corresponded to 22 DMGs (Refer **Supplementary Table 8**). Our final criterion was to choose a DMG that encompasses at least 3 DMPs, which led us to select the *SLFN12* gene as an epigenetic biomarker. *SLFN12* hosted 5 DMPs, distributed across TSS1500, TSS200, and the 1st Exon, and all exhibited methylation levels below 0.2 in HT cases (**Figure 7B**). Post identification of the epigenetic biomarker gene, we assessed the mRNA levels of the *SLFN12* gene using whole blood from 30 matched pairs of HT patients and healthy controls. The results revealed a significant elevation of *SLFN12* in HT patients (3.882 ± 1.355 vs. 1.026 ± 0.083, P < 0.001) (**Figure 7C**). **Figures 7D–H** illustrate the correlation between DNAm and *SLFN12* gene mRNA expression in 10 matched pairs of HT patients and healthy controls. The data suggest a negative correlation between the DNAm levels of *SLFN12* and its expression across all 5 DMPs in the 850K BeadChip (all P < 0.05). The area under the curve (AUC) value of 0.85 (sensitivity: 0.7, specificity: 0.7) for the 5 DMPs mapped to *SLFN12*, a potential biomarker, among HT patients compared to healthy controls, indicates the excellence of our model (**Figure 7I**). ROC curves were also constructed for each DMP and the AUC for all DMPs exceeded 0.85 (Refer **Supplementary Table 9**).

### 3.5 Correlations between SLFN12 DNA methylation levels and age, as well as thyroid function

**Figure 8:**
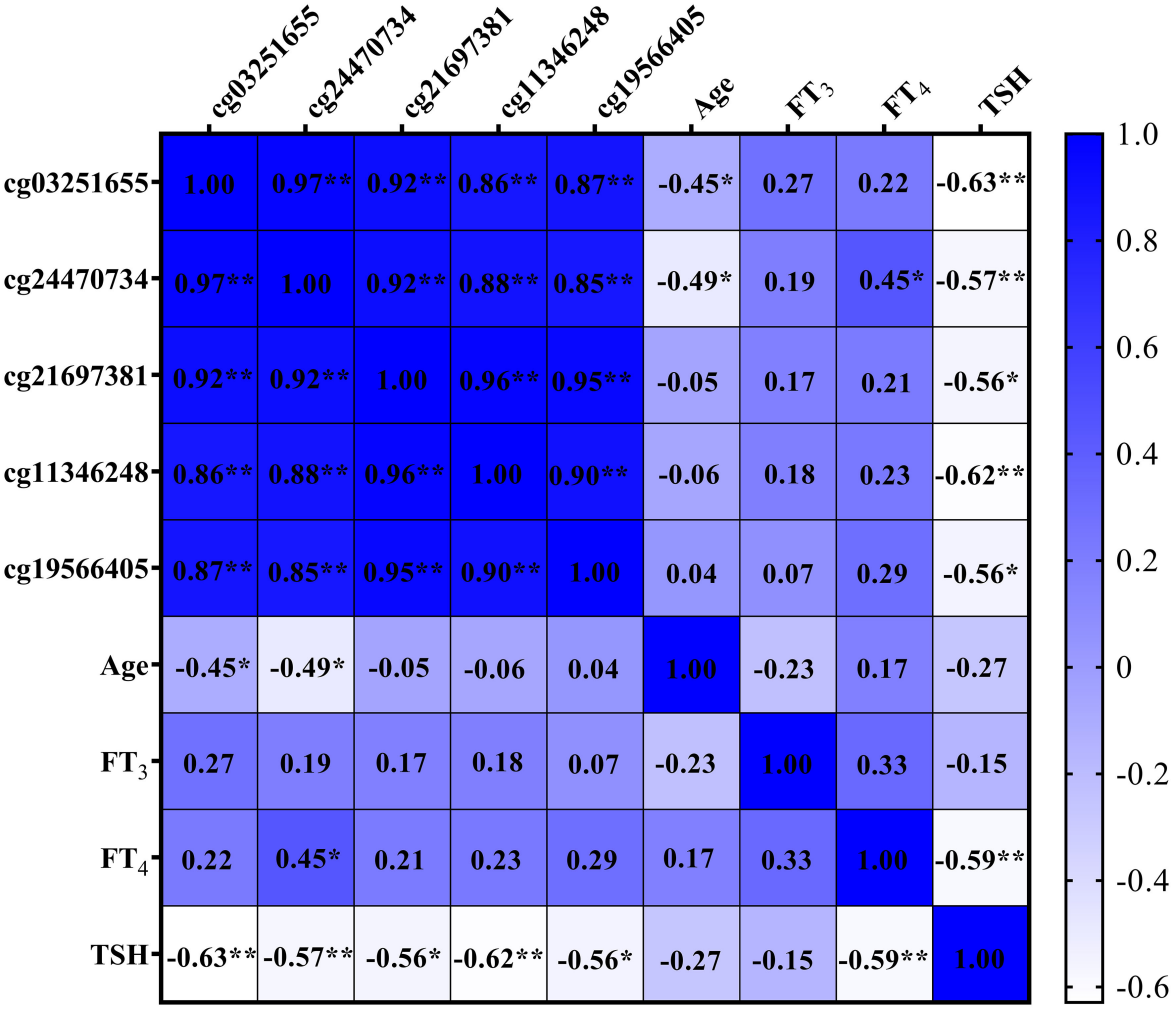
Heat map of correlations among SLFN12 DNA methylation levels, age, and thyroid function. * denotes P < 0.050; ** denotes P < 0.010.

**Figure 8** displays negative correlations between the DNAm levels of two DMPs situated in the *SLFN12* gene (cg03251655 and cg24470734) and age (both P < 0.050). Furthermore, all DMPs in the *SLFN12* gene show negative correlations with TSH levels (all P < 0.050). Additionally, one DMP (cg24470734) in the *SLFN12* gene demonstrates a positive correlation with FT_4_ levels (P < 0.050).

## 4 Discussion

Employing the Epigenome-wide association study (EWAS) is a commonly adopted approach for unearthing biomarkers within populations and for elucidating the molecular underpinnings of disease susceptibility. The 850K BeadChip serves as one of the instruments used in this methodology (11). In recent years, epigenetics has provided a potential link between genetics and autoimmune thyroid disease phenotype with some studies reporting a relationship between changes in DNAm patterns of some genes and HT (12). However, to the best of our knowledge, this is the first comprehensive study providing distinct DNAm profiles of HT patients screened from the general population based on EWAS. The 850K BeadChip we used was able to cover more than 850,000 CpGs, which greatly has advanced our understanding of the association between DNAm and HT, and demonstrated the importance of DNAm modification in the development of HT.

Prior research on a genome-wide scale has suggested alterations in the DNAm profiles in autoimmune disease patients (13). Several investigations have demonstrated that a characteristic trait of autoimmune diseases, including systemic lupus erythematosus, multiple sclerosis, and chronic spontaneous urticaria, is global hypomethylation (14). Our study findings align with these conclusions. Specifically, we found that in relation to the genome-wide DNAm levels (719,455 CpGs), the levels in patients with HT were significantly lower than in healthy individuals. Additionally, our findings indicated that despite a decrease in DNAm in other gene regions, the DNAm was heightened in promoter regions in HT patients, a result that corroborates many previous research conclusions (15). From these observations, it can be concluded that DNAm anomalies occur in the entire genome of HT patients. This is a key consideration for future HT research and may have practical implications in a clinical setting.

Initially, at the level of DMPs, our study observed distinct methylation patterns in HT, consisting of 283 DMPs that significantly deviated from healthy controls. Heatmap investigations and PCA results suggest that these DMPs serve as distinguishing features between HT patients and healthy controls, offering valuable theoretical basis for the continued exploration of HT and epigenetics. Subsequently, past research has demonstrated that the distribution of DMPs within the promoter region varies across diseases. In autoimmune diseases, a predominant characteristic is hypomethylated sites in the promoter region DMPs (16, 17), a finding that is reinforced by our research. Contrastingly, in carcinoma, hypermethylated sites are more prevalent within promoter region DMPs (18, 19). This indicates that the role of hypomethylation sites in the promoter region in autoimmune diseases’ pathogenesis warrants further investigation. Moreover, existing literature reveals a connection between chromosomal abnormalities and DNAm (19), implying that defining the distribution and properties of DMPs across various chromosomes can enhance disease comprehension. Our study discovered that the 283 DMPs in HT patients were extensively dispersed across autosomal chromosomes, with their count varying greatly across different chromosomes. The maximum number of DMPs (30 sites) were located on chromosome 6, and the most DMGs (15 genes) were found on chromosome 12. Interestingly, both chromosome 6 and chromosome 12 contained the DMG related to autoimmune diseases. On chromosome 6, the *HLA-DPB1* gene, a crucial gene in T lymphocyte activation and antigen presentation, is listed among the HT susceptibility genes (20). Concurrently, the C1R gene on chromosome 12 has been linked to the development of systemic autoimmune diseases (21, 22). Therefore, the distribution of DMPs across the chromosomes of HT patients is neither random nor uniform, suggesting that chromosomes with multiple DMPs or DMGs should be the focus in subsequent HT studies.

In our KEGG pathway analysis, we found that pathways involving cell adhesion molecules (hsa04514), Th17 cell differentiation (hsa04659), and the calcium signaling pathway (hsa04020) warrant careful consideration, given their established links with autoimmune diseases. Firstly, pathways associated with cell adhesion molecules are key to the adherence, rolling, and migration of leukocytes in inflamed tissues. Cell adhesion molecules specifically influence the migration of Th17 to inflammation sites, which effectively modulates immune response (23, 24). Secondly, recent findings underscore the critical role of Th17 cells, and their cellular and secretory components in the pathogenesis and progression of HT. Numerous studies have reported an elevated proportion of Th17 cells in HT patients (25, 26). In this context, the enrichment of the *SMAD3* gene in this pathway is noteworthy. Known for its vital role in autoimmunity (27, 28), *SMAD3* can control the activation of the TGF-β receptor, thereby influencing the differentiation of T cells into Th17 cells, and ultimately impacting the immune response. Thirdly, recent research has highlighted the crucial role of calcium signaling pathways in autoimmune diseases and inflammatory reactions. Kaufmann et al. demonstrated that calcium influx is a critical modulator of mitochondrial function and oxidative stress in Th17 cell-mediated multiorgan autoimmune disease and inflammation (29). Further, it has been shown that in several autoimmune diseases, B cell receptor calcium signaling exhibits defects, rendering B cells pathogenic and leading to the development of autoimmune diseases (30). While these pathways have been linked to autoimmune diseases, previous studies have not explored potential abnormalities in the methylation patterns of these pathways. Our research suggests that alterations in the methylation levels of key genes within these pivotal pathways might be implicated in HT pathogenesis.

Utilizing the state-of-the-art high-methylation BeadChip, we were able to filter a significant number of DMGs. The primary objective of our research was to pinpoint the crucial epigenetic biomarker gene, utilizing the data from the 850K BeadChip. Given the vast number of DMGs and the lack of a universally agreed approach to select epigenetic biomarker genes, we first excluded all DMPs that were not within the promoter region, given the ongoing debate over the influence of methylation sites outside the promoter region on gene expression (31). Further, DMPs with DNAm levels exceeding 0.7 or falling below 0.3 were selected, as such extreme levels are more likely indicative of lost expression or abundant expression. This approach aids in the discovery of potential biomarkers with functional relevance to disease phenotypes (16). Lastly, prior research has established that methylation patterns of multiple DMPs on promoters constitute key mechanisms influencing gene expression (15). Through these stringent criteria, we identified *SLFN12* as a potential epigenetic biomarker gene for HT. The *SLFN* family, discovered by Schwarz in 1988, encompasses genes that induce T cell cycle arrest, thus deactivating T cells (32). Fortunately, numerous studies have explored the relationship between *SLFN12* methylation and autoimmune diseases and inflammatory responses. Evidence suggests elevated *SLFN12* methylation levels in CD4+ and CD8+ T cells in the peripheral blood of patients with multiple sclerosis, a chronic inflammatory disease of the central nervous system. Such changes to *SLFN12* methylation pattern are thought to affect the activation and inflammatory response of T cells (33). In another study, researchers observed an increase in *SLFN12* gene methylation levels in the peripheral blood of patients with allergic rhinitis, with methylation levels correlating positively with inflammation severity, suggesting *SLFN12’s* association with immune response and inflammation (34). In this research, we identified five significant hypomethylated DMPs in the *SLFN12* gene promoter region in HT patients. Although this diverges from the hypermethylation pattern demonstrated in *SLFN12* in other inflammatory diseases, the shift to a hypomethylation pattern in HT patients substantially affected the expression of the *SLFN12* gene, showing a significant negative correlation. Additionally, a biomarker panel encompassing all discovered DMPs (especially cg11346248) on genes involving *SLFN12* exhibited outstanding diagnostic potential, accurately differentiating between HT patients and healthy controls. Since thyroid function is a crucial HT disease indicator, this article also seeks to establish the precise correlation between *SLFN12* methylation levels and thyroid function. Relevant research has suggested that genetic factors contribute to interindividual variations in FT_4_, FT_3_ and TSH levels (35). In 2021, a cohort study identified six CpG sites linked to elevated levels of FT_3_, and two CpG sites associated with elevated levels of TSH (36). Our findings indicated that methylation levels of all DMPs in the *SLFN12* gene were negatively correlated with TSH. Notably, one CpG site (cg24470734) had the largest |Δβ| among all CpG sites, and its methylation levels were positively correlated with FT_4_. Therefore, this finding implies that cg24470734 could have a significant impact on the link between thyroid function indicators. Consequently, we surmised that *SLFN12* gene methylation levels could affect thyroid function and predict changes in HT’s thyroid function. As a result, *SLFN12* may have a vital role in HT pathogenesis and its methylation pattern might serve as a potential epigenetic marker for HT. Nevertheless, these conclusions necessitate further validation through expansion of the sample size and more rigorous experimental evidence.

The strengths of our investigation are multifaceted. Ours is the pioneering study to undertake a genome-wide methylation analysis in HT, thus laying a robust theoretical groundwork for the involvement of epigenetics in HT. Additionally, the novelty of our research lies in the fact that for the first time, biological processes and pathways related to HT were evaluated from a methylation standpoint using bioinformatic analysis. This enabled the identification of potential methylation biomarkers for HT, and their transcriptional levels were subsequently verified. Nonetheless, it is important to recognize the limitations in our current study. A primary shortcoming of our study is that we only performed DNA methylation profiling on leukocytes present in the whole blood. To gain a more detailed understanding of the epigenetic changes associated with HT, conducting cell type-specific analysis after whole blood samples sequencing would be beneficial. A secondary limitation of our study pertains to the relatively small sample size. In future studies, a broader participant representation is required to corroborate our findings. Additionally, we will further investigate the evolution of *SLFN12* gene methylation levels in HT patients over time and disease progression, as well as explore the relationship between *SLFN12* gene methylation levels and HT treatment.

## 5 Conclusion

In essence, this investigation lays the foundation for an initial genome-wide DNAm profile for patients with HT, facilitating a clear distinction between HT subjects and healthy individuals from an epigenetic standpoint. Modifications in the methylation landscape of key genes, orchestrating processes such as cell adhesion molecules, calcium signaling pathways, and Th17 cell differentiation, may hold implications for HT pathogenesis. The gene *SLFN12* emerges as a potential epigenetic marker for HT and could bear substantial relevance to the disease’s progression. The comprehensive data yielded from this investigation offer significant insights, thereby enhancing our comprehension of aberrant DNAm contributing to HT’s pathogenesis.

## Data availability statement

The datasets presented in this study can be found in online repositories. The names of the repository/repositories and accession number(s) can be found below: BioProject accession number: PRJNA1005791.

## Ethics statement

The studies involving humans were approved by Harbin Medical University Ethics Review Committee (No. hrbmuecdc20200320). The studies were conducted in accordance with the local legislation and institutional requirements. The participants provided their written informed consent to participate in this study.

## Author contributions

ZZ: Conceptualization, Data curation, Investigation, Methodology, Software, Writing – original draft. JL: Writing – review & editing, Conceptualization, Data curation, Investigation, Software. YC: Writing – review & editing, Data curation, Investigation, Software, Supervision, Validation. BR: Data curation, Investigation, Methodology, Software, Writing – review & editing. SW: Conceptualization, Investigation, Writing – review & editing. YC: Writing – review & editing, Methodology, Supervision. YH: Validation, Writing – review & editing. QW: Software, Validation, Writing – review & editing. HG: Validation, Writing – review & editing. LL: Supervision, Validation, Visualization, Writing – review & editing. HS: Funding acquisition, Project administration, Resources, Supervision, Validation, Writing – review & editing.
