# Supplementary material for: Genome-wide DNA methylation pattern in whole blood of patients with Hashimoto thyroiditis
Source: Front Endocrinol (Lausanne). 2023 Nov 24;14:1259903. doi: 10.3389/fendo.2023.1259903 (PMC10704911; doi:10.3389/fendo.2023.1259903)
Supplement: Supplementary file 1 [file Table_1.docx]

**Supplementary table1 Demographic characteristics in HT patients and healthy controls**

|  |  | HT | Control |
| --- | --- | --- | --- |
| N |  | 30 | 30 |
| Sex | Female/male | 26/4 | 26/4 |
| Age (years) | Mean±SD | 44±13 | 44±12 |
| BMI (kg/m^2^ ) | Mean±SD | 24.61±4.22 | 24.92±3.05 |
| TSH (mIU/L) | M (P_25_–P_75_) | 2.55 (1.91–3.50)^a^ | 2.11 (1.73–2.64) |
| FT_3_ (pmol/L) | M (P_25_–P_75_) | 5.40 (4.99–5.71) | 5.38 (5.16–5.75) |
| FT_4_ (pmol/L) | M (P_25_–P_75_) | 15.99 (14.97–18.36) | 15.86 (13.99–17.15) |

HT, [Hashimoto thyroiditis](javascript:;); BMI, body mass index; TSH, thyroid stimulating hormone; FT_3_, free triiodothyronine; FT_4_, free thyroxine; a, indicates significant differences between HT and control groups, *P* <0.05.
