# Supplementary material for: Genome-wide DNA methylation pattern in whole blood of patients with Hashimoto thyroiditis
Source: Front Endocrinol (Lausanne). 2023 Nov 24;14:1259903. doi: 10.3389/fendo.2023.1259903 (PMC10704911; doi:10.3389/fendo.2023.1259903)
Supplement: Supplementary file 2 [file Table_2.docx]

**Supplementary table 2 Top 10 hyper- and hypo- DMPs in HT patients**

|  | **Probe** | **Gene** | **Chr** | **Location** | **Gene region** | **Δβ** | ***P* value** |
| --- | --- | --- | --- | --- | --- | --- | --- |
| Hypermethylation DMPs | cg02398342 | TBCD | 17 | 82750756 | TSS1500 | 0.389 | <0.001 |
|  | cg09293560 | REPIN1 | 7 | 150371151 | TSS200 | 0.343 | 0.008 |
|  | cg07599136 | AHRR | 5 | 415770 | Body | 0.266 | 0.019 |
|  | cg17429870 | BRSK2 | 11 | 1442432 | Body | 0.252 | 0.001 |
|  | cg17416644 | BRSK2 | 11 | 1453611 | Body | 0.244 | 0.001 |
|  | cg00809820 | TBCD | 17 | 82750637 | TSS1500 | 0.235 | 0.003 |
|  | cg21109666 | DISC1 | 1 | 231872095 | Body | 0.233 | 0.007 |
|  | cg25880954 | MGC12982 | 1 | 47434958 | TSS1500 | 0.225 | 0.019 |
|  | cg12502577 | NXPH4 | 12 | 57221798 | Body | 0.220 | 0.013 |
|  | cg10590925 | BRSK2 | 11 | 1442390 | Body | 0.214 | 0.007 |
| Hypomethylation DMPs | cg15108650 | TMEM168 | 7 | 112420018 | Body | -0.355 | 0.020 |
|  | cg08624915 | AHSP | 16 | 31538718 | TSS1500 | -0.339 | 0.009 |
|  | cg15465743 | BRSK2 | 11 | 1413145 | Body | -0.303 | 0.002 |
|  | cg17658113 | BRSK2 | 11 | 1413282 | Body | -0.235 | 0.005 |
|  | cg08041188 | KLRC4-KLRK1 | 12 | 10564015 | TSS1500 | -0.230 | 0.049 |
|  | cg04531182 | KLRC4-KLRK1 | 12 | 10563981 | TSS1500 | -0.229 | 0.045 |
|  | cg26708920 | FRMD4A | 10 | 13826317 | Body | -0.215 | 0.020 |
|  | cg01062020 | SH2D1B | 1 | 162382848 | TSS1500 | -0.194 | 0.026 |
|  | cg21517022 | GAREM | 18 | 30030173 | Body | -0.190 | 0.006 |
|  | cg17132030 | SLC27A1 | 19 | 17599784 | Body | -0.188 | 0.020 |

Chr, Chromosome; DMPs, differentially methylated positions; Δβ = The methylation level of case- The methylation level of control.
