# Supplementary material for: Genome-wide DNA methylation pattern in whole blood of patients with Hashimoto thyroiditis
Source: Front Endocrinol (Lausanne). 2023 Nov 24;14:1259903. doi: 10.3389/fendo.2023.1259903 (PMC10704911; doi:10.3389/fendo.2023.1259903)
Supplement: Supplementary file 3 [file Table_3.docx]

**Supplementary table 3 Chromosome distribution of DMGs**

| Chr | Hypomethylated DMGs | Hypermethylated DMGs |
| --- | --- | --- |
| 1 | ESPN, PHACTR4, RABGAP1L, SH2D1B | DISC1, EIF4G3, MGC12982,PBXIP1, SAMD11, SSBP3-AS1, UBXN11 |
| 2 | BOK, CHRND, ESPNL, HECW2, MYO7B | DOCK10, EPAS1, PPP3R1 |
| 3 | LINC00636, MBNL1 | ARL8B, BBX, STAC |
| 4 | ENPP6, GPM6A, PF4, SORCS2 | HTRA3, TACR3 |
| 5 | CPLX2, MCC, MYO10 | SLC6A19, SERINC5, AHRR, NLN |
| 6 | CASC15, GRM1, HLA-DPB1, KIAA0319, TMEM63B, TRIM10 | KIF25, TMEM151B, ZFAND3 |
| 7 | AUTS2, DNAJB6, GLCCI1, TMEM168,VIPR2,WBSCR26 | HOXA5, PTPRN2, REPIN1, YWHAG |
| 8 | ARHGEF10 | FLJ43860, SLC7A2 |
| 9 | PAX5, PDCD1LG2, PRUNE2 | FPGS, TNFSF8 |
| 10 | CACNB2, FRMD4A, PHYHIPL | AFAP1L2, FAM170B-AS1, GPRIN2, PALD1 |
| 11 | BRSK2, DRD4, LOC441601, OR5B12 | BRSK2, C11orf53, CYB5R2, KCNQ1DN, KLHL35, LDHC, MICAL2, PHF21A |
| 12 | C1R, CACNA1C, TESPA1, KLRC4-KLRK1, ITFG2-AS1, PIP4K2C, PWP1 | CCDC62, CDK17, CUX2, ING4, FAM234B, MGST1, NXPH4, SBNO1 |
| 13 | LINC00545, PRHOXNB, SOHLH2 | ATP8A2 |
| 14 | CDC42BPB, OR4K17 | FAM30A |
| 15 | EDC3, GNB5, MAP2K5, MTHFS, PHGR1, TPM1 | GREM1, SCG5, SMAD3 |
| 16 | AHSP, ANKRD11, HBA2, RLTPR | GRIN2A, MGRN1, MRPL28, MRPS34 |
| 17 | ASIC2, RAP1GAP2, SLFN12 | CHRNE, FN3K, RAI1, TBCD |
| 18 | GAREM, MYO5B, PIEZO2, PTPRM |  |
| 19 | ANKRD27, SLC27A1 | APC2, CFD, CRTC1, CYP2A7, DMPK, GNA11, MAG, TTYH1 |
| 20 | MYT1 | BCAS4, CDH4 |
| 21 | KRTAP12-3 |  |
| 22 | C22orf34 | LINC01399 |

Chr, Chromosome; DMGs, differentially methylated genes.
