# Supplementary material for: Genome-wide DNA methylation pattern in whole blood of patients with Hashimoto thyroiditis
Source: Front Endocrinol (Lausanne). 2023 Nov 24;14:1259903. doi: 10.3389/fendo.2023.1259903 (PMC10704911; doi:10.3389/fendo.2023.1259903)
Supplement: Supplementary file 4 [file Table_4.docx]

**Supplementary table 4 59 significant** biological process terms

| **ID** | **Description** | ***P* value** | **Gene ID** | **Count** |
| --- | --- | --- | --- | --- |
| GO:0042391 | Regulation of membrane potential | 1.18×10^-6^ | ASIC2/BOK/CACNA1C/CACNB2/CHRND/CHRNE/CRTC1/CUX2/DMPK/DRD4/GNA11/GRIN2A/GRM1/PIEZO2 | 14 |
| GO:0060078 | Regulation of postsynaptic membrane potential | 4.43×10^-5^ | CHRND/CHRNE/CUX2/DMPK/DRD4/GRIN2A/GRM1 | 7 |
| GO:1901385 | Regulation of voltage-gated calcium channel activity | 8.62×10^-5^ | CACNB2/DRD4/GNB5/STAC | 4 |
| GO:0099565 | Chemical synaptic transmission, postsynaptic | 9.25×10^-5^ | CHRND/CHRNE/CUX2/DMPK/DRD4/GRIN2A | 6 |
| GO:0006941 | Striated muscle contraction | <0.001 | ATP8A2/CACNA1C/CACNB2/CHRND/DMPK/STAC/TPM1 | 7 |
| GO:0003009 | Skeletal muscle contraction | <0.001 | ATP8A2/CHRND/DMPK/STAC | 4 |
| GO:0050804 | Modulation of chemical synaptic transmission | <0.001 | CACNB2/CPLX2/CRTC1/CUX2/DISC1/DMPK/DRD4/GRIN2A/GRM1/SORCS2/YWHAG | 11 |
| GO:0099177 | Regulation of trans-synaptic signaling | <0.001 | CACNB2/CPLX2/CRTC1/CUX2/DISC1/DMPK/DRD4/GRIN2A/GRM1/SORCS2/YWHAG | 11 |
| GO:0031346 | Positive regulation of cell projection organization | <0.001 | ANKRD27/ATP8A2/AUTS2/CDH4/CRTC1/CUX2/DISC1/ESPN/GPM6A/CARMIL2 | 10 |
| GO:0006584 | Catecholamine metabolic process | <0.001 | DRD4/EPAS1/GRIN2A/TACR3 | 4 |
| GO:0009712 | Catechol-containing compound metabolic process | <0.001 | DRD4/EPAS1/GRIN2A/TACR3 | 4 |
| GO:0050879 | Multicellular organismal movement | <0.001 | ATP8A2/CHRND/DMPK/STAC | 4 |
| GO:0050881 | Musculoskeletal movement | <0.001 | ATP8A2/CHRND/DMPK/STAC | 4 |
| GO:0050773 | Regulation of dendrite development | 0.001 | ANKRD27/CRTC1/CUX2/DISC1/HECW2/KIAA0319 | 6 |
| GO:0060079 | Excitatory postsynaptic potential | 0.001 | CHRND/CHRNE/CUX2/DMPK/GRIN2A | 5 |
| GO:0002027 | Regulation of heart rate | 0.001 | CACNA1C/CACNB2/EPAS1/TACR3/TPM1 | 5 |
| GO:0006936 | Muscle contraction | 0.001 | ATP8A2/CACNA1C/CACNB2/CHRND/CHRNE/DMPK/STAC/TACR3/TPM1 | 9 |
| GO:0046847 | Filopodium assembly | 0.001 | ESPN/GPM6A/MYO10/TTYH1 | 4 |
| GO:0003012 | Muscle system process | 0.001 | ATP8A2/CACNA1C/CACNB2/CHRND/CHRNE/DMPK/SMAD3/STAC/TACR3/TPM1 | 10 |
| GO:0010769 | Regulation of cell morphogenesis involved in differentiation | 0.001 | ANKRD27/BRSK2/CDH4/CUX2/DISC1/HECW2/KIAA0319/MAG | 8 |
| GO:0016358 | Dendrite development | 0.001 | ANKRD27/CRTC1/CUX2/DISC1/DOCK10/HECW2/KIAA0319 | 7 |
| GO:0043087 | Regulation of GTPase activity | 0.001 | ANKRD27/APC2/ARHGEF10/DOCK10/GNB5/KLRC4-KLRK1/PTPRN2/RABGAP1L/RAP1GAP2/TBCD | 10 |
| GO:0048675 | Axon extension | 0.001 | AUTS2/CDH4/DISC1/KIAA0319/MAG | 5 |
| GO:0022604 | Regulation of cell morphogenesis | 0.002 | ANKRD27/BRSK2/CDH4/CUX2/DISC1/HECW2/KIAA0319/MAG/MYO10/TPM1 | 10 |
| GO:0007015 | Actin filament organization | 0.002 | ARHGEF10/ESPN/ESPNL/MICAL2/MYO5B/MYO7B/CARMIL2/SMAD3/TPM1 | 9 |
| GO:1901019 | Regulation of calcium ion transmembrane transporter activity | 0.003 | CACNB2/DRD4/GNB5/STAC | 4 |
| GO:0006874 | Cellular calcium ion homeostasis | 0.003 | BOK/CACNA1C/CACNB2/DISC1/DMPK/DRD4/GRIN2A/GRM1/SMAD3 | 9 |
| GO:1903169 | Regulation of calcium ion transmembrane transport | 0.003 | CACNA1C/CACNB2/DRD4/GNB5/STAC | 5 |
| GO:0030048 | Actin filament-based movement | 0.003 | CACNA1C/CACNB2/MYO5B/MYO7B/TPM1 | 5 |
| GO:0055074 | Calcium ion homeostasis | 0.004 | BOK/CACNA1C/CACNB2/DISC1/DMPK/DRD4/GRIN2A/GRM1/SMAD3 | 9 |
| GO:0051017 | Actin filament bundle assembly | 0.004 | ARHGEF10/ESPN/ESPNL/SMAD3/TPM1 | 5 |
| GO:0030516 | Regulation of axon extension | 0.004 | CDH4/DISC1/KIAA0319/MAG | 4 |
| GO:1903522 | Regulation of blood circulation | 0.004 | ASIC2/CACNA1C/CACNB2/DMPK/EPAS1/TACR3/TPM1 | 7 |
| GO:0061572 | Actin filament bundle organization | 0.004 | ARHGEF10/ESPN/ESPNL/SMAD3/TPM1 | 5 |
| GO:0051494 | Negative regulation of cytoskeleton organization | 0.004 | APC2/ESPN/KIF25/CARMIL2/TBCD | 5 |
| GO:0070588 | Calcium ion transmembrane transport | 0.004 | CACNA1C/CACNB2/DRD4/GNB5/GPM6A/GRIN2A/STAC | 7 |
| GO:0120034 | Positive regulation of plasma membrane bounded cell projection assembly | 0.005 | AUTS2/ESPN/GPM6A/CARMIL2 | 4 |
| GO:0072503 | Cellular divalent inorganic cation homeostasis | 0.005 | BOK/CACNA1C/CACNB2/DISC1/DMPK/DRD4/GRIN2A/GRM1/SMAD3 | 9 |
| GO:0043547 | Positive regulation of GTPase activity | 0.005 | ANKRD27/APC2/ARHGEF10/DOCK10/GNB5/RABGAP1L/RAP1GAP2/TBCD | 8 |
| GO:0050806 | Positive regulation of synaptic transmission | 0.005 | CACNB2/CRTC1/CUX2/DRD4/GRIN2A | 5 |
| GO:1990138 | Neuron projection extension | 0.005 | AUTS2/CDH4/DISC1/KIAA0319/MAG | 5 |
| GO:0050954 | Sensory perception of mechanical stimulus | 0.006 | ASIC2/ESPN/ESPNL/MYO7B/PIEZO2 | 5 |
| GO:0018958 | Phenol-containing compound metabolic process | 0.006 | DRD4/EPAS1/GRIN2A/TACR3 | 4 |
| GO:0010639 | Negative regulation of organelle organization | 0.006 | ANKRD27/APC2/BOK/ESPN/KIF25/PAX5/CARMIL2/TBCD | 8 |
| GO:0061387 | Regulation of extent of cell growth | 0.006 | CDH4/DISC1/KIAA0319/MAG | 4 |
| GO:0045926 | Negative regulation of growth | 0.006 | GREM1/ING4/KIAA0319/MAG/RAI1/SMAD3 | 6 |
| GO:0008016 | Regulation of heart contraction | 0.007 | CACNA1C/CACNB2/DMPK/EPAS1/TACR3/TPM1 | 6 |
| GO:0008361 | Regulation of cell size | 0.007 | CDH4/DISC1/KIAA0319/MAG/RAP1GAP2 | 5 |
| GO:0120032 | Regulation of plasma membrane bounded cell projection assembly | 0.007 | AUTS2/ESPN/GPM6A/MYO10/CARMIL2 | 5 |
| GO:0045785 | Positive regulation of cell adhesion | 0.007 | DISC1/HLA-DPB1/TESPA1/KLRC4-KLRK1/MYO10/PDCD1LG2/SMAD3/TPM1 | 8 |
| GO:0060491 | Regulation of cell projection assembly | 0.007 | AUTS2/ESPN/GPM6A/MYO10/CARMIL2 | 5 |
| GO:2001257 | Regulation of cation channel activity | 0.007 | CACNB2/DRD4/GNB5/GRIN2A/STAC | 5 |
| GO:0050770 | Regulation of axonogenesis | 0.007 | BRSK2/CDH4/DISC1/KIAA0319/MAG | 5 |
| GO:0032412 | Regulation of ion transmembrane transporter activity | 0.008 | CACNB2/DRD4/GNB5/GRIN2A/HECW2/STAC | 6 |
| GO:1904062 | Regulation of cation transmembrane transport | 0.008 | CACNA1C/CACNB2/DRD4/GNB5/GRIN2A/HECW2/STAC | 7 |
| GO:0030218 | Erythrocyte differentiation | 0.008 | AHSP/EPAS1/HOXA5/TRIM10 | 4 |
| GO:0048167 | Regulation of synaptic plasticity | 0.008 | CPLX2/CRTC1/GRIN2A/SORCS2/YWHAG | 5 |
| GO:0048568 | Embryonic organ development | 0.009 | ATP8A2/DNAJB6/EPAS1/HOXA5/MICAL2/PAX5/PHACTR4/SMAD3 | 8 |
| GO:0022898 | Regulation of transmembrane transporter activity | 0.009 | CACNB2/DRD4/GNB5/GRIN2A/HECW2/STAC | 6 |

GO, Gene Ontology
