# Supplementary material for: Genome-wide DNA methylation pattern in whole blood of patients with Hashimoto thyroiditis
Source: Front Endocrinol (Lausanne). 2023 Nov 24;14:1259903. doi: 10.3389/fendo.2023.1259903 (PMC10704911; doi:10.3389/fendo.2023.1259903)
Supplement: Supplementary file 5 [file Table_5.docx]

**Supplementary table 5 10 significant** **cellular component** terms

| **ID** | **Description** | ***P* value** | **Gene ID** | **Count** |
| --- | --- | --- | --- | --- |
| GO:0099634 | Postsynaptic specialization membrane | <0.001 | CACNA1C/CHRND/CHRNE/GRIN2A/GRM1/SORCS2 | 6 |
| GO:0030027 | Lamellipodium | <0.001 | APC2/CDC42BPB/MCC/MYO10/PHACTR4/PTPRM/CARMIL2 | 7 |
| GO:0019897 | Extrinsic component of plasma membrane | 0.001 | APC2/CDH4/GNA11/GNB5/CARMIL2/STAC | 6 |
| GO:0031252 | Cell leading edge | 0.002 | APC2/CDC42BPB/MCC/MYO10/PHACTR4/PTPRM/CARMIL2/TACR3/TPM1 | 9 |
| GO:0005833 | Hemoglobin complex | 0.003 | AHSP/HBA2 | 2 |
| GO:1990454 | L-type voltage-gated calcium channel complex | 0.003 | CACNA1C/CACNB2 | 2 |
| GO:0099572 | Postsynaptic specialization | 0.003 | CACNA1C/CHRND/CHRNE/CRTC1/DISC1/GRIN2A/GRM1/SORCS2 | 8 |
| GO:0098839 | Postsynaptic density membrane | 0.003 | CACNA1C/GRIN2A/GRM1/SORCS2 | 4 |
| GO:0098858 | Actin-based cell projection | 0.004 | ESPN/ESPNL/GPM6A/MYO10/MYO7B/TTYH1 | 6 |
| GO:0031234 | Extrinsic component of cytoplasmic side of plasma membrane | 0.004 | GNA11/GNB5/CARMIL2/STAC | 4 |

GO, Gene Ontology.
