# Supplementary material for: Genome-wide DNA methylation pattern in whole blood of patients with Hashimoto thyroiditis
Source: Front Endocrinol (Lausanne). 2023 Nov 24;14:1259903. doi: 10.3389/fendo.2023.1259903 (PMC10704911; doi:10.3389/fendo.2023.1259903)
Supplement: Supplementary file 6 [file Table_6.docx]

**Supplementary table 6 13 significant molecular function terms**

| **ID** | **Description** | ***P* value** | **Gene ID** | **Count** |
| --- | --- | --- | --- | --- |
| GO:0005261 | cation channel activity | <0.001 | ASIC2/CACNA1C/CACNB2/CHRND/CHRNE/GPM6A/GRIN2A/PIEZO2/TMEM63B | 9 |
| GO:0022836 | gated channel activity | 0.001 | ASIC2/CACNA1C/CACNB2/CHRND/CHRNE/GRIN2A/PIEZO2/TMEM63B/TTYH1 | 9 |
| GO:0051015 | actin filament binding | 0.001 | CACNB2/ESPN/ESPNL/MYO10/MYO5B/MYO7B/TPM1 | 7 |
| GO:0030898 | actin-dependent ATPase activity | 0.001 | MYO10/MYO5B/MYO7B | 3 |
| GO:0005216 | ion channel activity | 0.001 | ASIC2/CACNA1C/CACNB2/CHRND/CHRNE/GPM6A/GRIN2A/PIEZO2/TMEM63B/TTYH1 | 10 |
| GO:0030594 | neurotransmitter receptor activity | 0.001 | CHRND/CHRNE/DRD4/GRIN2A/GRM1 | 5 |
| GO:0000146 | microfilament motor activity | 0.001 | MYO10/MYO5B/MYO7B | 3 |
| GO:0098960 | postsynaptic neurotransmitter receptor activity | 0.001 | CHRND/CHRNE/DRD4/GRM1 | 4 |
| GO:0015267 | channel activity | 0.002 | ASIC2/CACNA1C/CACNB2/CHRND/CHRNE/GPM6A/GRIN2A/PIEZO2/TMEM63B/TTYH1 | 10 |
| GO:0022803 | passive transmembrane transporter activity | 0.002 | ASIC2/CACNA1C/CACNB2/CHRND/CHRNE/GPM6A/GRIN2A/PIEZO2/TMEM63B/TTYH1 | 10 |
| GO:0022839 | ion gated channel activity | 0.003 | ASIC2/TMEM63B/TTYH1 | 3 |
| GO:0003779 | actin binding | 0.003 | CACNB2/ESPN/ESPNL/MICAL2/MYO10/MYO5B/MYO7B/PHACTR4/TPM1 | 9 |
| GO:0033218 | amide binding | 0.005 | GRIN2A/HLA-DPB1/MAG/MGST1/MTHFS/NLN/PPP3R1/VIPR2 | 8 |

GO, Gene Ontology
