# Supplementary material for: Genome-wide DNA methylation pattern in whole blood of patients with Hashimoto thyroiditis
Source: Front Endocrinol (Lausanne). 2023 Nov 24;14:1259903. doi: 10.3389/fendo.2023.1259903 (PMC10704911; doi:10.3389/fendo.2023.1259903)
Supplement: Supplementary file 7 [file Table_7.docx]

**Supplementary table 7 DMPs located in promoters**

| **Probe** | **Chr** | **Gene** | **Gene region** | **Δβ** | **Adjust *P*** | **Type** |
| --- | --- | --- | --- | --- | --- | --- |
| cg15657704 | 10 | AFAP1L2 | TSS1500 | 0.100 | 0.011 | Hyper |
| cg08624915 | 16 | AHSP | TSS1500 | -0.339 | 0.009 | Hypo |
| cg27413643 | 19 | ANKRD27 | 5'UTR | -0.130 | 0.038 | Hypo |
| cg19570618 | 3 | BBX | 5'UTR | 0.130 | 0.021 | Hyper |
| cg14094927 | 10 | CACNB2 | 5'UTR | -0.108 | 0.002 | Hypo |
| cg04912563 | 12 | CDK17 | 5'UTR | 0.106 | 0.009 | Hyper |
| cg22276371 | 2 | CHRND | TSS200 | -0.102 | 0.013 | Hypo |
| cg05875017 | 2 | CHRND | TSS200 | -0.111 | 0.013 | Hypo |
| cg15301006 | 5 | CPLX2 | 5'UTR | -0.107 | 0.011 | Hypo |
| cg25752163 | 12 | CUX2 | TSS1500 | 0.106 | 0.002 | Hyper |
| cg26468395 | 1 | EIF4G3 | 5'UTR | 0.120 | 0.015 | Hyper |
| cg17170839 | 4 | GPM6A | 1stExon | -0.114 | 0.002 | Hypo |
| cg00661861 | 10 | GPRIN2 | 5'UTR | 0.143 | 0.034 | Hyper |
| cg04938149 | 16 | HBA2 | TSS1500 | -0.112 | 0.005 | Hypo |
| cg04863892 | 7 | HOXA5 | TSS200 | 0.122 | 0.049 | Hyper |
| cg25203481 | 11 | KCNQ1DN | TSS200 | 0.103 | 0.002 | Hyper |
| cg00944873 | 6 | KIAA0319 | TSS1500 | -0.172 | 0.027 | Hypo |
| cg25486399 | 12 | KIAA0748 | 5'UTR | -0.161 | 0.000 | Hypo |
| cg25108548 | 12 | KIAA1467 | TSS1500 | 0.152 | 0.009 | Hyper |
| cg04531182 | 12 | KLRC4-KLRK1 | TSS1500 | -0.229 | 0.045 | Hypo |
| cg08041188 | 12 | KLRC4-KLRK1 | TSS1500 | -0.230 | 0.049 | Hypo |
| cg14105637 | 21 | KRTAP12-3 | TSS200 | -0.102 | 0.000 | Hypo |
| cg11821245 | 11 | LDHC | TSS200 | 0.180 | 0.029 | Hyper |
| cg07398106 | 11 | LDHC | 5'UTR | 0.137 | 0.044 | Hyper |
| cg06584561 | 13 | LINC00545 | TSS1500 | -0.114 | 0.043 | Hypo |
| cg00259046 | 11 | LOC441601 | TSS200 | -0.137 | 0.004 | Hypo |
| cg25880954 | 1 | MGC12982 | TSS1500 | 0.225 | 0.019 | Hyper |
| cg10548102 | 12 | MGST1 | TSS1500 | 0.101 | 0.009 | Hyper |
| cg08296601 | 16 | MRPL28 | 5'UTR | 0.150 | 0.026 | Hyper |
| cg17422692 | 16 | MRPL28 | 5'UTR | 0.156 | 0.030 | Hyper |
| cg08923669 | 16 | MRPL28 | 5'UTR | 0.140 | 0.049 | Hyper |
| cg13126979 | 16 | MRPS34 | TSS1500 | 0.128 | 0.011 | Hyper |
| cg04124281 | 15 | MTHFS | TSS1500 | -0.127 | 0.045 | Hypo |
| cg09703048 | 14 | OR4K17 | TSS1500 | -0.113 | 0.032 | Hypo |
| cg11741455 | 11 | OR5B12 | TSS1500 | -0.110 | 0.000 | Hypo |
| cg07237217 | 10 | PALD1 | 5'UTR | 0.163 | 0.022 | Hyper |
| cg23216745 | 1 | PBXIP1 | TSS1500 | 0.204 | 0.037 | Hyper |
| cg11299543 | 9 | PDCD1LG2 | 1stExon | -0.107 | 0.040 | Hypo |
| cg15398841 | 4 | PF4 | TSS200 | -0.100 | 0.012 | Hypo |
| cg02530824 | 4 | PF4 | TSS200 | -0.131 | 0.014 | Hypo |
| cg16072462 | 4 | PF4 | TSS200 | -0.100 | 0.022 | Hypo |
| cg06834998 | 4 | PF4 | TSS200 | -0.112 | 0.022 | Hypo |
| cg05509609 | 4 | PF4 | TSS200 | -0.121 | 0.036 | Hypo |
| cg12699599 | 15 | PHGR1 | TSS200 | -0.106 | 0.029 | Hypo |
| cg25133016 | 12 | PIP4K2C | TSS1500 | -0.101 | 0.032 | Hypo |
| cg25345738 | 12 | PWP1 | TSS1500 | -0.177 | 0.034 | Hypo |
| cg00632811 | 12 | PWP1 | TSS1500 | -0.135 | 0.046 | Hypo |
| cg10140454 | 17 | RAI1 | 5'UTR | 0.129 | 0.038 | Hyper |
| cg09293560 | 7 | REPIN1 | TSS200 | 0.343 | 0.008 | Hyper |
| cg15744134 | 15 | SCG5 | TSS200 | 0.103 | 0.008 | Hyper |
| cg01062020 | 1 | SH2D1B | TSS1500 | -0.194 | 0.026 | Hypo |
| cg21501207 | 1 | SH2D1B | TSS1500 | -0.180 | 0.034 | Hypo |
| cg26333902 | 8 | SLC7A2 | 1stExon | 0.129 | 0.046 | Hyper |
| cg03251655 | 17 | SLFN12 | 1stExon | -0.171 | 0.001 | Hypo |
| cg24470734 | 17 | SLFN12 | TSS200 | -0.178 | 0.002 | Hypo |
| cg21697381 | 17 | SLFN12 | TSS1500 | -0.117 | 0.002 | Hypo |
| cg11346248 | 17 | SLFN12 | TSS1500 | -0.102 | 0.002 | Hypo |
| cg19566405 | 17 | SLFN12 | TSS1500 | -0.116 | 0.002 | Hypo |
| cg02486855 | 15 | SMAD3 | TSS1500 | 0.108 | 0.018 | Hyper |
| cg23731272 | 15 | SMAD3 | TSS1500 | 0.125 | 0.027 | Hyper |
| cg18538958 | 4 | TACR3 | 1stExon | 0.107 | 0.039 | Hyper |
| cg02398342 | 17 | TBCD | TSS1500 | 0.389 | 0.000 | Hyper |
| cg00809820 | 17 | TBCD | TSS1500 | 0.235 | 0.003 | Hyper |
| cg05496603 | 6 | TMEM151B | TSS1500 | 0.157 | 0.001 | Hyper |
| cg17240725 | 7 | WBSCR26 | TSS200 | -0.125 | 0.036 | Hypo |

Chr, Chromosome; DMPs, differentially methylated positions; Δβ = The methylation level of case- The methylation level of control; Hypo, hypomethylation; Hyper, hypermethylation.
