# Supplementary material for: Genome-wide DNA methylation pattern in whole blood of patients with Hashimoto thyroiditis
Source: Front Endocrinol (Lausanne). 2023 Nov 24;14:1259903. doi: 10.3389/fendo.2023.1259903 (PMC10704911; doi:10.3389/fendo.2023.1259903)
Supplement: Supplementary file 8 [file Table_8.docx]

**Supplementary table 8 DMPs and DMGs with absolute high or low methylation level**

| **Probe** | **Chr** | **Gene** | **Gene region** | **Case mean** | **Control mean** | **Δβ** | **Adjust *P*** | **Type** |
| --- | --- | --- | --- | --- | --- | --- | --- | --- |
| cg27413643 | 19 | ANKRD27 | 5'UTR | 0.098 | 0.228 | -0.130 | 0.038 | Hypo |
| cg04912563 | 12 | CDK17 | 5'UTR | 0.899 | 0.793 | 0.106 | 0.009 | Hyper |
| cg05875017 | 2 | CHRND | TSS200 | 0.198 | 0.309 | -0.111 | 0.013 | Hypo |
| cg04863892 | 7 | HOXA5 | TSS200 | 0.712 | 0.590 | 0.122 | 0.049 | Hyper |
| cg04531182 | 12 | KLRC4-KLRK1 | TSS1500 | 0.272 | 0.501 | -0.229 | 0.045 | Hypo |
| cg11821245 | 11 | LDHC | TSS200 | 0.776 | 0.596 | 0.180 | 0.029 | Hyper |
| cg07398106 | 11 | LDHC | 5'UTR | 0.955 | 0.818 | 0.137 | 0.044 | Hyper |
| cg06584561 | 13 | LINC00545 | TSS1500 | 0.730 | 0.844 | -0.114 | 0.043 | Hypo |
| cg25880954 | 1 | MGC12982 | TSS1500 | 0.956 | 0.731 | 0.225 | 0.019 | Hyper |
| cg04124281 | 15 | MTHFS | TSS1500 | 0.256 | 0.383 | -0.127 | 0.045 | Hypo |
| cg09703048 | 14 | OR4K17 | TSS1500 | 0.297 | 0.410 | -0.113 | 0.032 | Hypo |
| cg11741455 | 11 | OR5B12 | TSS1500 | 0.755 | 0.864 | -0.110 | 0.000 | Hypo |
| cg23216745 | 1 | PBXIP1 | TSS1500 | 0.912 | 0.708 | 0.204 | 0.037 | Hyper |
| cg11299543 | 9 | PDCD1LG2 | 1stExon | 0.281 | 0.388 | -0.107 | 0.040 | Hypo |
| cg12699599 | 15 | PHGR1 | TSS200 | 0.144 | 0.250 | -0.106 | 0.029 | Hypo |
| cg25133016 | 12 | PIP4K2C | TSS1500 | 0.087 | 0.188 | -0.101 | 0.032 | Hypo |
| cg25345738 | 12 | PWP1 | TSS1500 | 0.195 | 0.372 | -0.177 | 0.034 | Hypo |
| cg00632811 | 12 | PWP1 | TSS1500 | 0.147 | 0.282 | -0.135 | 0.046 | Hypo |
| cg01062020 | 1 | SH2D1B | TSS1500 | 0.214 | 0.407 | -0.194 | 0.026 | Hypo |
| cg21501207 | 1 | SH2D1B | TSS1500 | 0.105 | 0.285 | -0.180 | 0.034 | Hypo |
| cg03251655 | 17 | SLFN12 | 1stExon | 0.194 | 0.365 | -0.171 | 0.001 | Hypo |
| cg24470734 | 17 | SLFN12 | TSS200 | 0.141 | 0.319 | -0.178 | 0.002 | Hypo |
| cg21697381 | 17 | SLFN12 | TSS1500 | 0.145 | 0.262 | -0.117 | 0.002 | Hypo |
| cg11346248 | 17 | SLFN12 | TSS1500 | 0.109 | 0.211 | -0.102 | 0.002 | Hypo |
| cg19566405 | 17 | SLFN12 | TSS1500 | 0.112 | 0.228 | -0.116 | 0.002 | Hypo |
| cg02486855 | 15 | SMAD3 | TSS1500 | 0.906 | 0.799 | 0.108 | 0.018 | Hyper |
| cg23731272 | 15 | SMAD3 | TSS1500 | 0.892 | 0.767 | 0.125 | 0.027 | Hyper |
| cg18538958 | 4 | TACR3 | 1stExon | 0.298 | 0.191 | 0.107 | 0.039 | Hyper |
| cg02398342 | 17 | TBCD | TSS1500 | 0.957 | 0.568 | 0.389 | 0.000 | Hyper |
| cg05496603 | 6 | TMEM151B | TSS1500 | 0.726 | 0.569 | 0.157 | 0.001 | Hyper |

Chr, Chromosome; DMPs, differentially methylated positions; DMGs, differentially methylated genes; Δβ = The methylation level of case- The methylation level of control; Hypo, hypomethylation; Hyper, hypermethylation.
