# Supplementary material for: Genome-wide DNA methylation pattern in whole blood of patients with Hashimoto thyroiditis
Source: Front Endocrinol (Lausanne). 2023 Nov 24;14:1259903. doi: 10.3389/fendo.2023.1259903 (PMC10704911; doi:10.3389/fendo.2023.1259903)
Supplement: Supplementary file 9 [file Table_9.docx]

**Supplementary table 9 ROC evaluation of DNA methylation levels at methylation sites found on *SLFN12* in patients with HT compared with controls**

| **Probe** | **AUC** | **Sensitivity** | **Specificity** | ***P* value** |
| --- | --- | --- | --- | --- |
| cg03251655 | 0.89 | 1 | 0.7 | 0.003 |
| cg24470734 | 0.88 | 1 | 0.7 | 0.004 |
| cg21697381 | 0.87 | 1 | 0.7 | 0.005 |
| cg11346248 | 0.91 | 1 | 0.7 | 0.002 |
| cg19566405 | 0.87 | 1 | 0.8 | 0.005 |
| All DMPs on *SLFN12* | 0.85 | 0.9 | 0.7 | < 0.001 |

ROC, receiver operating characteristic; AUC, area under curve.
